# Supplementary material for: From sequence to enzyme mechanism using multi-label machine learning
Source: BMC Bioinformatics. 2014 May 19;15:150. doi: 10.1186/1471-2105-15-150 (PMC4229970; doi:10.1186/1471-2105-15-150)
Supplement: Additional file 2 — Java code of ml2db. Additional file ml2db_code.tar.gz contains the Java source code to run the multi-label machine learning experiments and save the results to database. The code’s Javadoc is included. [file 1471-2105-15-150-S2.zip › additional file 2/ml2db/utils/doc/uk/ac/standrews/utils/main/FileUtils.html]

FileUtils


JavaScript is disabled on your browser.


- Overview
- Package
- Class
- Use
- Tree
- Deprecated
- Index
- Help

- Prev Class
- Next Class

- Frames
- No Frames

- All Classes

- Summary:
- Nested |
- Field |
- Constr |
- Method

- Detail:
- Field |
- Constr |
- Method


uk.ac.standrews.utils.main

## Class FileUtils

- java.lang.Object
- - uk.ac.standrews.utils.main.FileUtils

- ---

    

  ```
  public class FileUtils
  extends java.lang.Object
  ```

  Utils to read, write files etc.

  Version:
  :   24-Mar-2007

  Author:
  :   ldeferra

- - ### Constructor Summary

    Constructors

    Constructor and Description

    `FileUtils()`
  - ### Method Summary

    Methods

    | Modifier and Type | Method and Description |
    | --- | --- |
    | `static void` | `appendPropertiesToFile(java.io.File file, java.util.Properties properties)` |
    | `static void` | `appendToFile(java.io.File file, java.lang.String text)` Append text to file |
    | `static boolean` | `appendToFile(java.lang.String filePath, java.lang.String text)` Append text to file |
    | `static void` | `appendToFileAndPrint(java.lang.String filePath, java.lang.String text)` Append print text to screen (system.out) and append it to file. |
    | `static boolean` | `canReadFile(java.io.File file)` Tests wether the file can be read |
    | `static boolean` | `canReadFile(java.lang.String path)` Tests whether the file can be read |
    | `static boolean` | `canReadParent(java.lang.String path)` Checks whether a file directory is readable. |
    | `static boolean` | `canWriteFile(java.io.File file)` Tests whether the file can be written |
    | `static boolean` | `canWriteFile(java.lang.String path)` Tests wether the file can be written |
    | `static boolean` | `canWriteParent(java.lang.String path)` Checks whether a file directory is writable. |
    | `static boolean` | `deleteFile(java.lang.String fileName)` Delete a file |
    | `static void` | `fileNotWritablePopUp(java.lang.String filePath)` Error Pop-up: file not writable |
    | `static long` | `fileSize(java.lang.String filePath)` Get a file size (length in bytes) |
    | `static java.lang.String[][]` | `fileToArrayOfArrays(java.lang.String fileName, java.lang.String regExpOfSeparator, boolean discardFirstLine)` Reads a special-character-separated file (eg comma or tab separated) and creates an array of rows-columns. |
    | `static java.util.ArrayList<java.lang.String>` | `fileToArrayOfLines(java.io.File file)` Reads a file and adds the lines to an array, adapted from: http://www.computing.net/programming/wwwboard/forum/12276.html |
    | `static java.util.ArrayList<java.lang.String>` | `fileToArrayOfLines(java.lang.String filePath)` Reads a file and adds the lines to an array, adapted from: http://www.computing.net/programming/wwwboard/forum/12276.html |
    | `static java.util.ArrayList<java.lang.String>` | `fileTokensToArray(java.lang.String fileName, java.lang.String regexp)` Reads a file and adds the tokens (identified by regular expressions) to an array (e.g. |
    | `static void` | `fromArrayOfArraysToFile(java.util.ArrayList<java.util.ArrayList<java.lang.String>> lists, java.lang.String fileName, java.lang.String regExpOfSeparator)` Writes a special-character-separated file (eg comma or tab separated) and fills it from an array of rows-columns. |
    | `static java.io.BufferedReader` | `fromFilePathToBufferedReader(java.lang.String filePath)` From file path to bufferedReader |
    | `static java.lang.String[]` | `getDirectoryFiles(java.lang.String directoryPath)` Gets the list of files in a directory From http://www.faqs.org/docs/javap/c10/s2.html |
    | `static java.lang.String` | `getDirectoryPath(java.lang.String path)` Get the parent of a full file path (directory + filename) |
    | `static java.lang.String` | `getFileNameFromDirectory(java.lang.String directoryPath, java.lang.String pattern)` Get the first file name in the directory matching the given pattern string. |
    | `static java.lang.String` | `getFileNameFromFullPath(java.lang.String path)` Get the filename from a full file path (directory + filename) |
    | `static long` | `getFileSizeInKb(java.lang.String filePath)` Get the file size in Kb |
    | `static java.util.zip.GZIPInputStream` | `getGunzipInputStream(java.io.BufferedInputStream source)` |
    | `static java.io.File` | `getPotentialDirectory(java.io.File file)` Get a directory out of a path |
    | `static java.lang.String` | `getStringFromBufferedReader(java.io.BufferedReader reader)` |
    | `static java.lang.String` | `getStringFromFile(java.lang.String filePath)` Get all the text in a file as a single string |
    | `static java.util.zip.GZIPInputStream` | `gunzipFile(java.io.File inputFile)` |
    | `static int` | `gunzipFile2(java.io.File file_input, java.io.File dir_output)` Gunzip the input archive. |
    | `static void` | `main(java.lang.String[] args)` Main method for testing |
    | `static void` | `printAllPaths(java.io.File file)` Test method to print all absolute/relative paths of a file |
    | `static void` | `printFromReader(java.io.BufferedReader reader)` Prints all lines from a reader. |
    | `static boolean` | `stringExistsInFile(java.lang.String filePath, java.lang.String string)` |
    | `static boolean` | `writeToFile(java.lang.String filePath, java.lang.String text)` |

    - ### Methods inherited from class java.lang.Object

      `equals, getClass, hashCode, notify, notifyAll, toString, wait, wait, wait`

- - ### Constructor Detail


    - #### FileUtils

      ```
      public FileUtils()
      ```
  - ### Method Detail


    - #### appendPropertiesToFile

      ```
      public static void appendPropertiesToFile(java.io.File file,
                                java.util.Properties properties)
      ```


    - #### appendToFile

      ```
      public static void appendToFile(java.io.File file,
                      java.lang.String text)
      ```

      Append text to file

      Parameters:
      :   `file` - a File object
      :   `text` - a text string

      Throws:
      :   `java.io.IOException`


    - #### appendToFile

      ```
      public static boolean appendToFile(java.lang.String filePath,
                         java.lang.String text)
      ```

      Append text to file

      Parameters:
      :   `filePath` - the full path to an existing file
      :   `text` - a text string


    - #### appendToFileAndPrint

      ```
      public static void appendToFileAndPrint(java.lang.String filePath,
                              java.lang.String text)
      ```

      Append print text to screen (system.out) and append it to file.

      Parameters:
      :   `filePath` - the full path to an existing file
      :   `text` - a text string


    - #### canReadFile

      ```
      public static boolean canReadFile(java.io.File file)
      ```

      Tests wether the file can be read


    - #### canReadFile

      ```
      public static boolean canReadFile(java.lang.String path)
      ```

      Tests whether the file can be read


    - #### canReadParent

      ```
      public static boolean canReadParent(java.lang.String path)
      ```

      Checks whether a file directory is readable. For example for file
      /home/user/file.txt, it checks if /home/user/ is readable

      Parameters:
      :   `path` - directory path + filename

      Returns:
      :   true if the path before the last slash is readable


    - #### canWriteFile

      ```
      public static boolean canWriteFile(java.io.File file)
      ```

      Tests whether the file can be written


    - #### canWriteFile

      ```
      public static boolean canWriteFile(java.lang.String path)
      ```

      Tests wether the file can be written


    - #### canWriteParent

      ```
      public static boolean canWriteParent(java.lang.String path)
      ```

      Checks whether a file directory is writable. For example for file
      /home/user/file.txt, it checks if /home/user/ is writable

      Parameters:
      :   `path` - directory path + filename

      Returns:
      :   true if the path before the last slash is writable


    - #### deleteFile

      ```
      public static boolean deleteFile(java.lang.String fileName)
      ```

      Delete a file

      Parameters:
      :   `filePath` - the full path to a file

      Returns:
      :   true if the file was deleted


    - #### fileNotWritablePopUp

      ```
      public static void fileNotWritablePopUp(java.lang.String filePath)
      ```

      Error Pop-up: file not writable


    - #### fileToArrayOfArrays

      ```
      public static java.lang.String[][] fileToArrayOfArrays(java.lang.String fileName,
                                             java.lang.String regExpOfSeparator,
                                             boolean discardFirstLine)
      ```

      Reads a special-character-separated file (eg comma or tab separated) and
      creates an array of rows-columns. Each column contains the column data
      elements

      Parameters:
      :   `fileName` - the full path to the file to be read
      :   `regExpOfSeparator` - the regular expression separating the tokens in the line (e.g.
          a comma for comma separated values files)

      Returns:
      :   an array of arrays in which each element is a token from the file
          (row by columns)


    - #### fileToArrayOfLines

      ```
      public static java.util.ArrayList<java.lang.String> fileToArrayOfLines(java.io.File file)
      ```

      Reads a file and adds the lines to an array, adapted from:
      http://www.computing.net/programming/wwwboard/forum/12276.html

      Parameters:
      :   `file` - the file to read from

      Returns:
      :   the array containing the file lines


    - #### fileToArrayOfLines

      ```
      public static java.util.ArrayList<java.lang.String> fileToArrayOfLines(java.lang.String filePath)
      ```

      Reads a file and adds the lines to an array, adapted from:
      http://www.computing.net/programming/wwwboard/forum/12276.html

      Parameters:
      :   `filePath` - the path of the file to read from

      Returns:
      :   the array containing the file lines


    - #### fileTokensToArray

      ```
      public static java.util.ArrayList<java.lang.String> fileTokensToArray(java.lang.String fileName,
                                                            java.lang.String regexp)
      ```

      Reads a file and adds the tokens (identified by regular expressions) to
      an array (e.g. for flat files with a long list of values \_without\_ any
      new-line) adapted from:
      http://www.computing.net/programming/wwwboard/forum/12276.html

      Parameters:
      :   `fileName` - the path of the file to read from

      Returns:
      :   array with the string tokens


    - #### fromArrayOfArraysToFile

      ```
      public static void fromArrayOfArraysToFile(java.util.ArrayList<java.util.ArrayList<java.lang.String>> lists,
                                 java.lang.String fileName,
                                 java.lang.String regExpOfSeparator)
                                          throws java.io.IOException
      ```

      Writes a special-character-separated file (eg comma or tab separated) and
      fills it from an array of rows-columns. Each column contains the column
      data elements

      Parameters:
      :   `fileName` - the file to be read
      :   `regExpOfSeparator` - the regular expression separating the tokens in the line (e.g.
          a comma for comma separated values files)

      Throws:
      :   `java.io.IOException`


    - #### fromFilePathToBufferedReader

      ```
      public static java.io.BufferedReader fromFilePathToBufferedReader(java.lang.String filePath)
      ```

      From file path to bufferedReader

      Parameters:
      :   `filePath` -

      Returns:
      :   a BufferedReader

      Throws:
      :   `java.io.FileNotFoundException`


    - #### getDirectoryFiles

      ```
      public static java.lang.String[] getDirectoryFiles(java.lang.String directoryPath)
      ```

      Gets the list of files in a directory From
      http://www.faqs.org/docs/javap/c10/s2.html

      Parameters:
      :   `directoryPath` -

      Returns:
      :   a list of files in the directory


    - #### getDirectoryPath

      ```
      public static java.lang.String getDirectoryPath(java.lang.String path)
      ```

      Get the parent of a full file path (directory + filename)

      Parameters:
      :   `path` - a full file path (directory + filename)

      Returns:
      :   the parent directory full path


    - #### getFileNameFromDirectory

      ```
      public static java.lang.String getFileNameFromDirectory(java.lang.String directoryPath,
                                              java.lang.String pattern)
      ```

      Get the first file name in the directory matching the given pattern
      string.

      Parameters:
      :   `pattern` - a regular expression

      Returns:
      :   the path of the first file matching the pattern


    - #### getFileNameFromFullPath

      ```
      public static java.lang.String getFileNameFromFullPath(java.lang.String path)
      ```

      Get the filename from a full file path (directory + filename)

      Parameters:
      :   `path` - a full file path (directory + filename)

      Returns:
      :   the filename (string after the last slash or backslash)


    - #### getFileSizeInKb

      ```
      public static long getFileSizeInKb(java.lang.String filePath)
      ```

      Get the file size in Kb


    - #### getGunzipInputStream

      ```
      public static java.util.zip.GZIPInputStream getGunzipInputStream(java.io.BufferedInputStream source)
      ```


    - #### getPotentialDirectory

      ```
      public static java.io.File getPotentialDirectory(java.io.File file)
      ```

      Get a directory out of a path


    - #### getStringFromBufferedReader

      ```
      public static java.lang.String getStringFromBufferedReader(java.io.BufferedReader reader)
      ```


    - #### getStringFromFile

      ```
      public static java.lang.String getStringFromFile(java.lang.String filePath)
      ```

      Get all the text in a file as a single string

      Parameters:
      :   `filePath` -

      Returns:
      :   all the text in the file as a single string

      Throws:
      :   `java.io.IOException`


    - #### gunzipFile

      ```
      public static java.util.zip.GZIPInputStream gunzipFile(java.io.File inputFile)
      ```


    - #### gunzipFile2

      ```
      public static int gunzipFile2(java.io.File file_input,
                    java.io.File dir_output)
      ```

      Gunzip the input archive. Send the output to the directory specified by
      dir\_output. Assumes that the input file name ends with ".gz" from
      http://www
      .particle.kth.se/~lindsey/JavaCourse/Book/Part1/Supplements/Chapter09
      /compressDecompress.html


    - #### main

      ```
      public static void main(java.lang.String[] args)
      ```

      Main method for testing

      Parameters:
      :   `args` -

      Throws:
      :   `java.sql.SQLException`


    - #### printAllPaths

      ```
      public static void printAllPaths(java.io.File file)
                                throws java.io.IOException
      ```

      Test method to print all absolute/relative paths of a file

      Parameters:
      :   `file` -

      Throws:
      :   `java.io.IOException`


    - #### printFromReader

      ```
      public static void printFromReader(java.io.BufferedReader reader)
                                  throws java.io.IOException
      ```

      Prints all lines from a reader.

      Parameters:
      :   `reader` - a buffered reader object (taking text from a file, or URL
          etc.)

      Throws:
      :   `java.io.IOException`


    - #### stringExistsInFile

      ```
      public static boolean stringExistsInFile(java.lang.String filePath,
                               java.lang.String string)
      ```


    - #### writeToFile

      ```
      public static boolean writeToFile(java.lang.String filePath,
                        java.lang.String text)
      ```


    - #### fileSize

      ```
      public static long fileSize(java.lang.String filePath)
      ```

      Get a file size (length in bytes)

      Returns:


- Overview
- Package
- Class
- Use
- Tree
- Deprecated
- Index
- Help

- Prev Class
- Next Class

- Frames
- No Frames

- All Classes

- Summary:
- Nested |
- Field |
- Constr |
- Method

- Detail:
- Field |
- Constr |
- Method
